# Patients’ experiences of, and psychological responses to, surveillance for pulmonary nodules detected through lung cancer screening

**DOI:** 10.1136/bmjresp-2024-002498

**Published:** 2025-06-12

**Authors:** Evangelos Katsampouris, Andrew W. Creamer, Ruth Prendecki, Elizabeth Clark, Jennifer L. Dickson, Richard Lee, Samuel M. Janes, Stephen W. Duffy, Samantha L. Quaife

**Affiliations:** 1 Centre for Cancer Screening, Prevention and Early Diagnosis, Wolfson Institute of Population Health, Queen Mary University of London, London, UK; 2 Lungs for Living Research Centre, UCL Respiratory, Division of Medicine, University College London, London, UK; 3 School of Education, University of Aberdeen, Aberdeen, UK; 4 Lung Unit, The Royal Marsden NHS Foundation Trust, London, UK

**Keywords:** lung cancer

## Abstract

**Introduction:**

Low-dose CT screening reduces lung cancer mortality among high-risk populations, and detects indeterminate pulmonary nodules that require subsequent surveillance. This period of uncertainty could result in patients experiencing lung cancer-related distress, anxiety and worry. This multicentre qualitative study explored patients’ experiences and psychological responses to disclosing and communicating nodule surveillance.

**Methods:**

Eligible participants were purposively sampled from four lung cancer screening sites in England to ensure diversity with respect to region, service setting, individual characteristics and surveillance pathways. Thirty-nine patients (23 females), aged 55–80 years, who had undergone their first nodule surveillance scan, participated in one-to-one remote semi-structured interviews. Audio-recorded interviews were transcribed verbatim and analysed using applied thematic analysis.

**Results:**

Participants reported a broad spectrum of psychological responses to the way their nodule finding was communicated and their experiences of undergoing surveillance. Understanding what a nodule is and what a surveillance process entails was important for explaining patient psychological reactions and behavioural outcomes. Perceived support and effective communication with healthcare professionals were instrumental in decreasing patients’ distress, uncertainty and concern, and increasing reassurance, knowledge about nodules and psychological preparation for the possibility of surveillance.

**Conclusions:**

While current letter-based means of nodule disclosure and communication were acceptable to patients, there is a need to improve the way nodules are communicated using lay language. Brief verbal consultations with healthcare professionals could provide clearer guidance to patients undergoing surveillance and increase their understanding about the surveillance process and subsequent scans, resulting in improved affective, behavioural and cognitive outcomes.

**WHAT IS ALREADY KNOWN ON THIS TOPIC**

- Evidence has demonstrated the efficacy of low-dose CT in early lung cancer detection but highlighted challenges posed by detecting indeterminate pulmonary nodules, leading to psychological distress, worry and uncertainty.

**WHAT THIS STUDY ADDS**

- This study adds insights into patients’ psychological responses during surveillance for indeterminate pulmonary nodules, emphasising the role of effective communication and healthcare support in alleviating distress and uncertainty.

**HOW THIS STUDY MIGHT AFFECT RESEARCH, PRACTICE OR POLICY**

- This study underlines the necessity for improved communication in pulmonary nodule surveillance, potentially shaping healthcare practices in lung cancer screening communication protocols.

## Introduction

 Lung cancer (LC) has the highest cancer-related mortality worldwide.^1 2^ While survival is highly dependent on the stage of diagnosis (55% 5-year survival for people diagnosed with LC at stage 1 vs 5% 5-year survival at stage 4 in England),^3^ only 21.1% of people in England are diagnosed at stage 1 vs 49.4% at stage 4.^4^ Achieving earlier diagnosis is crucial to improving mortality.

Lung cancer screening (LCS) using low-dose CT(LDCT) detects LC at an early treatable stage among populations at high risk of LC (ie, aged 55–80 years with a significant and recent tobacco smoking history), usually before they experience symptoms. Consequently, LDCT screening reduces the risk of LC mortality—by 20% compared with chest X-ray in the National Lung Screening Trial and by 24% (men only) in the Dutch-Belgian trial ‘NELSON’ (Dutch acronym for ‘Nederlands-Leuvens Longkanker Screenings ONderzoek’).^5 6^ LDCT screening has been implemented in several countries (eg, the USA, Canada) and is recommended for national roll-out in the UK.

One potential barrier to the acceptability of LCS is the relatively high frequency of indeterminate pulmonary nodules; nodules of low but not negligible malignancy probability for which guidelines (including Lung-RADS^7^ and British Thoracic Society^8^) advise surveillance CT scan at 3–6 months. Across UK LCS trials, approximately 11% of participants had an indeterminate pulmonary nodule at baseline screening CT, which required surveillance.^9^ Previous research has found that individuals receiving indeterminate findings experience negative psychological outcomes during the months spent waiting for their first surveillance scan,^10 11^ although this appears to resolve in the longer term.^12 13^ The negative psychological outcomes can arise from individuals being placed in a prolonged period of uncertainty, concern and emotional distress,^1012 14^,^16^ with women, individuals experiencing socioeconomic deprivation, and those who currently smoke more likely to experience clinically elevated anxiety and LC-related distress while waiting for a surveillance CT.^1112 17^,^19^ Crucially, psychological distress or negative experiences of surveillance could adversely affect individuals’ subsequent screening behaviour, including adherence to surveillance or subsequent screening rounds,^20^,^22^ and satisfaction with screening.^23^,^26^ Indeed, research in the context of incidentally diagnosed nodules (ie, outside of screening) has highlighted patients’ emotional discomfort and prolonged uncertainty when they perceive their cancer-related concerns are not addressed by healthcare professionals (HCPs), which might lead to poor adherence to surveillance and inadequate knowledge of their care plan.^27 28^ Data from the USA have found that adherence to follow-up after a positive screening examination is low,^29^ and it is therefore essential that all these factors are addressed to ensure screen-detected findings are followed up appropriately.

Ensuring individuals considering screening are well-informed and aware of the frequency with which indeterminate nodules are detected^8^ and the possibility of needing surveillance has been suggested to reduce cancer-related distress among those receiving a nodule diagnosis.^10^ A series of studies in the US healthcare context of incidentally detected nodules suggests HCPs often underestimate the psychological impact these ‘near-cancer’ diagnoses have on patients.^23^ The framework by Slatore and Wiener argues that high-quality, patient-centred communication strategies are needed to mediate how patients respond, to promote adaptive psychological responses and support cancer knowledge and prevention behaviour during surveillance.^14 23^ However, there is little evidence of patients’ experiences, reactions and behaviours following the information and communication they receive when undergoing surveillance for an indeterminate nodule detected through screening, with no qualitative research to date in the UK LCS context.

This study aimed to explore patients’ experiences of and psychological responses to screen-detected indeterminate pulmonary nodule disclosure and surveillance after their first follow-up LDCT scan. The objectives were to explore patients’ psychological and behavioural reactions to screen-detected lung nodule disclosure, surveillance and communication; and to investigate modifiable aspects of patients’ experience to inform the development of evidence-based communication practices that could promote positive patient-centred outcomes.

## Methods

### Study design

Data were collected between June 2022 and February 2023. This multicentre qualitative study employed remote semi-structured interviews with English-speaking patient participants, aged 55–80 years. The study’s methods were reported in line with the COnsolidated criteria for REporting Qualitative research.^30^

### Participants

Eligible individuals were those aged 55–80 years who had undergone their first surveillance scan for an indeterminate pulmonary nodule detected by LDCT LCS at one of four LCS services in England, including one research trial in London (the SUMMIT Study, which aimed to assess the implementation of LDCT screening for LC in a high-risk population in North Central and East London and validate a multicancer early detection blood test)^31^ and three of NHS England’s Targeted Lung Health Check Programme^32^ sites located in Nottingham, Halton, Liverpool and Knowsley, and Stoke. Eligible individuals were identified locally at each LCS site from clinic lists, which included screen-detected pulmonary nodule surveillance patients.

Purposive sampling was used to ensure diversity with respect to region, service setting, individual characteristics (ie, smoking status, ethnicity, educational attainment, age, gender) and to represent three possible subsequent management decisions after the initial surveillance LDCT: (i) stable nodule volume requiring repeat LDCT scan at 12 months (negative), (ii) indeterminate changes requiring further surveillance CT at 3 months (indeterminate) and (iii) suspicious changes requiring urgent referral for diagnostic workup (unfavourable)^8^ in order to explore diverse patients’ experiences and responses to nodule communication and surveillance. Negative and indeterminate outcomes were communicated to patients mainly via written letter.

### Interviews

One-to-one, in-depth semi-structured interviews were conducted (by EK) remotely by telephone or Microsoft Teams and audio-recorded. The interview schedule (online supplemental file 1) was developed in consultation with patient representatives, respiratory clinicians and psychologists, and informed by the patient-centred communication conceptual framework.^14 23^ The interview schedule was used flexibly to ensure that all topics of interest were covered and any unprompted topics raised and prioritised by participants were explored. Interviewees were asked open-ended questions about their experiences, thoughts and feelings of their first lung nodule surveillance scan, their understanding of possible results and knowledge about nodules, the meaning of and expectations for surveillance, their experience of HCPs’ communication, their concerns about their nodule and any unmet needs and suggestions for additional support.

### Patient and public involvement

The research team sought the views of three patient representatives affected by LC, including one individual who was curatively treated for LC, one individual receiving palliative care for LC and one individual who lost their partner to LC. They agreed on the importance of the research question and key objectives likely to lead to patient benefit. They gave feedback on the appropriateness of the research design and provided their insights on the content of the interview schedule, agreeing that in-depth interviews would achieve a richer account of experiences and that it was acceptable to approach patients during surveillance. A patient representative/coauthor was involved throughout the duration of research, including attendance on the Study Management Group meetings so that any arising issues with the study could be resolved with their insights too, providing insightful input and constructive feedback on study materials, expressing their views and interpretation on pseudonymised results of the study and being involved in dissemination.

### Procedure

Eligible individuals were identified by clinical LCS staff via locally held patient records and the research site’s research (the SUMMIT Study) database, within 4 weeks of receiving the scan results of their first nodule surveillance appointment.

Potential participants were posted an invitation pack consisting of an invitation letter, participant information sheet, consent form, expression of interest form and a freepost envelope for reply. Interested individuals were asked to contact the research team directly via phone, email or letter. If potential participants met all mandatory inclusion criteria, they were asked to provide information about demographic and smoking characteristics (ie, gender, age, level of education, ethnicity, smoking group) using an ‘eligibility and entry characteristics form’, and verbal informed consent which was audio-recorded, prior to participating in the interview. Participants were offered a £25 voucher gift, which could be used in different online or physical stores, as a thank you for their time.

Participants were assured that their data would be kept confidential and pseudonymised. This was ensured by giving each participant an assigned pseudonym (unrelated sequence of characters) rather than including their names or any other personally identifiable data with the interview transcripts. Any personally identifiable data were password-protected and stored separately from pseudonymised interview transcripts on the University’s network drive. Only the researcher/interviewer had the master code linking any personally identifiable data with interview transcripts. Audio recordings of the interviews were deleted after the transcriptions had been checked for accuracy by the researcher.

### Analysis

Interviews were transcribed verbatim. Applied thematic analysis^33^ was used to explore patterns in the data being important to patients’ experiences, to inductively code and interpret themes within a communication conceptual framework,^23^ supported by NVivo (QSR International, V.12/2020). Familiarisation with the data began during the interviews and continued through repeated reading of the transcripts. Initially, all interview transcripts were read and coded by EK. Ten per cent of transcripts were independently coded by SLQ. EK and SLQ developed a codebook using the primary analytical aims, which consisted of wider codes of participants’ interview data (eg, thoughts and emotions about nodule findings, suggestions for improvement, patient-centred outcomes, perceived support, experiences of screening, experiences of communicating with HCPs).

This codebook (ie, wider codes) was analysed inductively in order to organise the results into lower-order and higher-order themes (eg, data from the wider codes of ‘thoughts and emotions about nodule findings’ and ‘experiences of screening’ were organised into the ‘reactions and outcomes’ theme). Themes were then independently interpreted and discussed, along with the use of the patient-centred communication conceptual framework.^23^ Although findings’ interpretation should have been influenced to an extent by the use of the communication framework, it was important to better understand the influences that affected patients’ perceptions of the current means in nodule communication and their involvement in the decision-making of the surveillance plan, and to explore any modifiable factors that impacted patients’ psychological and behavioural outcomes. The communication framework was used to inform the interview schedule and theme interpretation about how and why patient participants experienced nodule findings and surveillance as individuals and addressed their concerns in relation to HCPs’ communication practices.

Disagreements related to developing a codebook and themes were resolved via multiple opportunities for discussion and reflection in regular meetings until consensus was reached. They discussed and agreed on key themes, which were iterated at regular intervals alongside progress with the analysis.

To ensure rigour and trustworthiness in data, interpretation and methods, EK and SLQ reviewed, discussed and made some necessary minor amendments on the interview schedule after a few first interviews had been conducted and transcribed. This was important to reflect on the aim of the study, the qualitative methods that were employed to collect data on the topics of interest, the progress of recruitment and data collection, any support or further training EK (the interviewer) would need for analysis and to feed back on EK’s style and areas of improvement for conducting interviews. The research team held meetings with the Study Management Group, consisting of experienced research and clinical academics and a patient representative, to discuss and interpret some preliminary findings in line with the objectives and plan of analysis, and to reflect on any challenges faced during the interviews and any interesting points raised by patients.

## Results

Across all four LCS sites, 261 individuals were identified as eligible and invited to participate, of which 53 (20.3%) expressed their interest to participate and 39 (73.6%) individuals (M_age_=67.6 years, SD=5.6, range 58–79) (23 females) participated (M_duration_=35.5 min, SD=11.3, range 17–61). Following the initial surveillance LDCT, 30.8% of participants had a negative outcome, 46.2% had an indeterminate outcome and 23.1% of individuals did not provide researchers with their surveillance group. We were unable to recruit any individuals from the unfavourable outcome group. Most interviews were carried out by telephone (n=38) and one using Microsoft Teams (table 1).

**Table 1:** Participants’ demographic and smoking characteristics

	Lung cancer screening sites				Total
	Site 1(n=13)	Site 2(n=8)	Site 3(n=8)	Site 4(n=10)	N=39
Outcome of initial surveillance LDCT, n (%)					
Negative	6 (46.2)	3 (37.5)	2 (25.0)	1 (10.0)	12 (30.8)
Indeterminate	4 (30.8)	4 (50.0)	3 (37.5)	7 (70.0)	18 (46.2)
Not provided*	3 (23.1)	1 (12.5)	3 (37.5)	2 (20.0)	9 (23.1)
Gender, n (%)					
Male	2 (15.4)	4 (50.0)	4 (50.0)	6 (60.0)	16 (41.0)
Female	11 (84.6)	4 (50.0)	4 (50.0)	4 (40.0)	23 (59.0)
Highest level of education, n (%)					
Finished school at or before the age of 15 years	0	0	1 (12.5)	0	1 (2.6)
CSE/GCSE/O-level or equivalent	8 (61.5)	7 (87.5)	6 (75.0)	5 (50.0)	26 (66.7)
A-levels or equivalent	1 (7.7)	1 (12.5)	0	1 (10.0)	3 (7.7)
Higher education qualification below degree	1 (7.7)	0	0	2 (20.0)	3 (7.7)
Degree or higher degree	3 (23.1)	0	1 (12.5)	1 (10.0)	5 (12.8)
Prefer not to say	0	0	0	1 (10.0)	1 (2.6)
Ethnicity, n (%)					
White	12 (92.3)	8 (100)	8 (100)	10 (100)	38 (97.4)
Black	1 (7.7)	0	0	0	1 (2.6)
Smoking status, n (%)					
Current	2 (15.4)	1 (12.5)	3 (37.5)	5 (50.0)	11 (28.2)
Occasionally	3 (23.1)	0	0	1 (10.0)	4 (10.3)
Former	8 (61.8)	7 (87.5)	5 (62.5)	4 (40.0)	24 (61.5)
Age (years), M (SD)	68.6 (6.2)	67.9 (4.6)	67.4 (6.7)	66.3 (4.9)	67.6 (5.6)
Years since quit smoking, M (SD)	5.3 (6.3)	9.7 (17.7)	8.0 (13.1)	5.5 (8.7)	6.8 (11.1)

* Not provided by the interviewee.

LDCT, low-dose CT.

**Figure 1:**
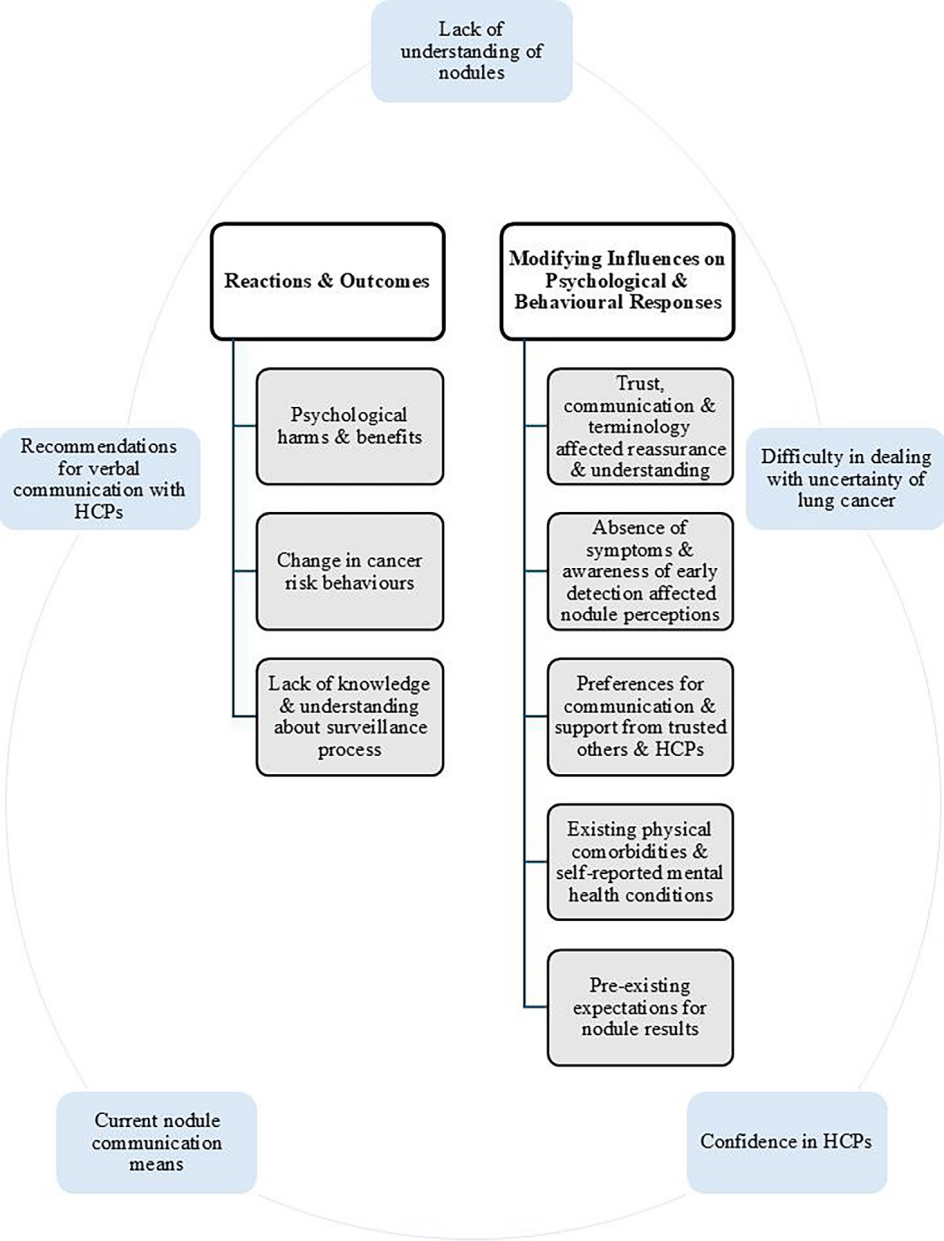
This figure shows the two higher-order themes along with their lower-order themes in relation to patients’ experiences and psychological responses to nodule disclosure and surveillance in the context of low-dose CT lung cancer screening. It also depicts five codes (in rectangular shape) that overlapped across the themes and influenced patients’ perceptions. HCP, healthcare professional.

Two higher-order themes, which were guided by the inductive analysis of the codebook (ie, wider codes), were used to organise the results of the data analysis: (1) psychological and behavioural reactions and outcomes; (2) modifying influences on psychological and behavioural responses. Theme descriptions are supported by longer illustrative quotes (eg, Q1/Q2) presented in table 2 and in online supplemental file 2. Figure 1 depicts the two higher-order themes along with their lower-order themes that appeared to affect patients’ experiences and reactions to nodule disclosure and surveillance in LDCT LC screening, and modifying influences of their psychological responses and perceptions. This figure also presents five codes (ie, lack of understanding of nodules, difficulty in dealing with uncertainty of LC, confidence in HCPs, current nodule communication means, recommendations for verbal communication with HCPs) that overlapped among themes, and participants described these in relation to their experiences, reactions and psychological outcomes. For example, recommendations for verbal communication with HCPs (code) were described along with their lack of knowledge and understanding about the surveillance process (lower-order theme), which resulted in affecting their preferences for communication and support from trusted others and HCPs (lower-order theme) as well as perceived reassurance and understanding (lower-order theme).

**Table 2:** Selective illustrative quotes for theme descriptions

Quote no	Higher-order themes	Lower-order themes	Quote
Q1	Reactions and outcomes	Psychological harms and benefits	*“Frightened, I suppose, because I don’t know, it’s a stupid thing to say, but once I’d got the results of the second scan and they’d told me what the plan of action was right now, you’re going, this is what’s going to happen, and it did, and I was scared and still in disbelief because I kept thinking no, no, no, no, no, they’ve got me mixed up, are you sure you haven’t got me mixed up with somebody else”? (F/FS*)
Q4		Change in cancer risk behaviours	*“Not really. I have a pair of lungs. They workish. I don’t cough. I now find it quite difficult to walk up the escalator at a tube station. I get out of breath more easily than I used to before. I am aware of that. But I can still swim, I still dance without any problem, without getting breathless. I’m fine, I think, for my age”. (F/CS*)
Q7		Lack of knowledge and understanding about the surveillance process	*“Yeah, I, I’m concerned about this delay, that it might not be good news. I’m, I find the longer I’m waiting for the results, the more negative I feel about it. Not the whole experience of taking part in the lung health check, but of the second scan and what they may have found. I’ve found that negative. If I’d have had the result by now then I’ve had, obviously it was, you’d know what you’re dealing with but I don’t know what I’m dealing with at the moment because I haven’t heard from them”. (F/FS*)
Q14	Modifying influences on psychological and behavioural responses	Trust, communication and terminology affected reassurance and understanding	*“At that time. I can, if I’ve got any other questions, the consultant, when I got called into the hospital to see him, he asked me a few times if I’ve got any questions, and I just kept saying no, because nothing come to mind. But he said, if you do think of something, don’t hesitate to phone my secretary and I’ll phone you back, or come and see me. But, no, I haven’t worried about it. I just leave it. He didn’t seem that concerned, so I thought, well, if he doesn’t seem that concerned, and I don’t have to go back until another 12 months”. (F/CS*)
Q19		Absence of symptoms and awareness of early detection affected nodule perceptions	*“It was just that something had been found and actually I was quite relieved because I thought well, if there is anything there it’s because I’ve got no, I’m not wheezy, I don’t cough. I don’t bring anything up. Apart from the fact that now I do get very, very breathless but at the time I had no symptoms at all. So I actually found it, that if there was anything untoward someone was aware of it and was keeping an eye on it. So yeah, so no it was fine”. (F/FS*)
Q21		Preferences for communication and support from trusted others and HCPs within and outside of the screening team	*“No, I, actually I thought it was very, very clear and it, I knew exactly what they were saying in the letter. There was no misunderstanding, I knew exactly. Actually, it was quite a very good letter explaining what was wrong, what they found and then the follow up scan. So, yeah, I was quite impressed with the letter. … No, because obviously receiving the letter, I was relieved, so I thought there was no reason to phone them up again”. (M/CS*)
Q36		Existing physical comorbidities and self-reported mental health conditions	*“… because I was already in the stage of, I’ve got COPD, so I knew there was a problem with my lung … Because I do suffer, I suffer with this breathlessness and sometimes it’s as if I’ve got no oxygen in my body, that type of a thing”. (M/FS*)
Q42		Pre-existing expectations for nodule results	*“Well, when I look at my previous lifestyle, like I said, when I was younger, I was a really heavy smoker, so I anticipated that had caused some damage to my lungs because of that activity. So, I was, I didn’t need anything explaining. I knew what was going on … I was a little, like I say, I’ve been a heavy smoker and heavy drinker in the past, so I'm expecting there to be some damage as a result of that. So, I wasn’t unduly nervous or anything. I was just curious as to why I needed to go back and later on after, they finally told me why, which was that they’d seen a third nodule. But I wasn’t upset or anything, but I was, knew what was going on”. (M/FS*)

Participant codes (eg, M/CS) represent gender (ie, M, F) and smoking status (ie, CS, FS).

COPD, chronic obstructive pulmonary disease; CS, currently smoking; F, female; FS, formerly smoked; HCP, healthcare professional; M, male.

### Reactions and outcomes

Participants reported a broad spectrum of positive and negative psychological responses to the finding of an indeterminate nodule, the way this finding was communicated and their experiences of undergoing surveillance. These were interpreted from participants’ and researchers’ analyses to have different affective/emotional, behavioural and cognitive outcomes.

### Psychological harms and benefits

Disclosure of screen-detected nodule findings evoked distress, anxiety, worry and concern about the risk of nodules being LC. Some participants reported feelings of fear and threat, being ‘shocked’, ‘nervous’, ‘in disbelief’ and ‘scared’ and finding it difficult to cope with the uncertainty of waiting during the surveillance period (Q1). In one case, a participant believed the result meant they had LC (Q2).

Others emphasised the relief from receiving their LDCT results despite these showing a nodule, and reassurance from being invited to regular repeat scan appointments. Confidence that HCPs ‘kept an eye on’ their nodule during the subsequent scans increased perceived support and satisfaction, to the extent that one participant described ‘looking forward’ to the surveillance scans (Q3).

### Change in cancer risk behaviours

Some participants emphasised that following the result, they attempted to maintain a better quality of life, adopted risk-reducing behaviours (eg, exercise) and were more positively minded to improve their lung health (Q4). These appeared to be important in increasing optimism towards their overall health and compensating for concerns about the impact of their smoking (Q5). However, some participants highlighted their inability to quit smoking, which led to increased worry implying that more effective cessation support is needed (Q6).

### Lack of knowledge and understanding about the surveillance process

Some participants spoke of their lack of knowledge or ‘not remembering’ why they had been invited to repeat scans. Difficulty in understanding the rationale for waiting several months for a surveillance scan led some participants to feel concerned and nervous for a long period of time about what the repeat scans would show. Some also made different inferences about the severity of their nodule based on their interpretations of the speed of the communication they received. Some participants who did not hear back in a timely manner considered that everything was fine, whereas others interpreted this to mean something negative about their result (Q7).

In a few cases, participants were looking not only for explanations about what a nodule is, but specifically guidance and support from HCPs on how concerned they should be, and crucially the reasons why this level of concern was low (Q8). Some described that it was not enough to state that there was ‘no need to worry’ and that it was crucial for this to be supported by a clear explanation of the nodule and why there is no immediate follow-up (Q9). They commented that this would prevent them from relying on potentially misleading information found online, or from needing to seek additional support from GPs or from significant others with similar experiences or knowledge (Q10/Q11/Q12/Q13).

### Modifying influences on psychological and behavioural responses

Participants explained the reasons influencing their experiences, psychological and emotional reactions and level of understanding related to undergoing nodule surveillance and the different communications and information exchanges with HCPs. They suggested how their expectations, needs for further support and experiences of surveillance could be improved.

### Trust, communication and terminology affected reassurance and understanding

The trust and confidence participants placed in HCPs and the reassurance they perceived from their interactions appeared to explain variation in how participants responded emotionally to surveillance. The extent to which participants felt reassured seemed to be guided by the level of concern they interpreted that their HCP had about their nodule (Q14/Q15). Specifically, the complexity, brevity, tone of reassurance and choice of phrasing for the nodule seemed to guide participants’ understanding and level of concern. The use of the words ‘abnormality’ or ‘anomaly’ to describe the nodule appeared to evoke fear among some participants about their lung health. Brief and concise wording reassured some, while for others, too little information about the nodule appeared to limit their understanding. They described how this lack of clarity increased uncertainty over what the next steps were (Q16/Q17). Nodule terminology that felt ambiguous was treated with suspicion, with some participants interpreting this ambiguity as an intentional strategy to mask the potential nodule severity and reduce participant anxiety (Q18).

### Absence of symptoms and awareness of early detection affected nodule perceptions

The absence of symptoms reassured some participants about the severity of the nodule, with some inferring that nothing could be seriously wrong (Q19). However, others appeared to be confused about how and why a nodule was detected when they have no symptoms, inferring ‘there is nothing to worry about’. Others held positive views that prevention is better than cure, highlighting that ‘early detection is the best prevention’, which appeared to motivate them to have repeat scans. Understanding the importance of early detection reassured participants about subsequent scans, with one participant stating that ‘early detection is absolutely key’ (Q20).

### Preferences for communication and support from trusted others and HCPs within and outside of the screening team

Participants held mixed beliefs about the means through which their nodule was communicated. Crucially, it appeared that when the communication they received was misaligned with their preferences, participants reported more adverse psychological responses. Some participants found their nodule results letter to be self-explanatory and serve its purpose if it was timely, and there were clearly signposted opportunities to seek information and support from HCPs (Q21/Q22/Q23). However, others described low awareness, lack of knowledge and difficulty in understanding what a nodule is through a brief letter, highlighting the need to improve the written explanation for nodules using lay language (Q24/Q25). Limited communication with HCPs increased anxiety and uncertainty over their surveillance plan and heightened concern about nodules being LC (Q26/Q27/Q28).

Together with being unable to ask HCPs further questions, some participants perceived there to be low levels of instrumental and emotional support from their family. However, for some participants, it was a conscious choice not to share this information with them because it was difficult to manage and cope with their reactions and ‘panic’ (Q29), although some significant others stated that it was a ‘good idea’ for participants to attend the repeat scans.

Some participants emphasised the importance of verbal communication with HCPs, over letters, to provide reassurance and improve their understanding of what a nodule is and the surveillance process (Q30/Q31/Q32). Participants also highlighted the importance of primary care’s involvement in supporting them, receiving a copy of the scan results and explaining the results (Q33/Q34). One participant expected GPs to be involved in the postscreening process as an adjunct tier of support, outside the hospital, that is more directly accessible than secondary care and provides continuity of care with their broader medical history (Q35).

### Existing physical comorbidities and self-reported mental health conditions

Participants with existing respiratory infections or comorbidities (eg, chronic obstructive pulmonary disease, asthma) viewed these conditions to be one of the main reasons their nodule was found. They felt that their nodule added to existing physical comorbidities and exacerbated other problems (eg, breathlessness), which interfered with daily activities (Q36/Q37/Q38). Additionally, being diagnosed with other cancer types (eg, breast cancer) appeared to increase worrying thoughts and difficulty in dealing with poor health (Q39). Others also described how existing mental health conditions (eg, depression) negatively impacted their ability to cope emotionally with the nodule and increased levels of anxiety and concern (Q40). One participant recognised the HCPs adapting their communication in response to their emotional state and mental health conditions to reduce any negative impact of communicating the nodule (Q41).

### Pre-existing expectations for nodule results

Participants expressed their prior perceptions of their lung health when explaining how they reacted to their nodule finding (Q42). Most assumed and expected an abnormal screening result because of their long-term smoking and/or because they worked in mines (Q43/Q44). They emphasised pragmatism in their attitude, describing how it was only a matter of time before a nodule was found due to ageing and past risk behaviours. They were, though, grateful for being invited to surveillance scans, and one participant described feeling ‘happy’ about the nodule because it was not LC.

## Discussion

To our knowledge, this is the first UK study to explore how patients experience and react to indeterminate pulmonary nodule disclosure and surveillance in the LDCT LCS context. Participants reported both positive and negative psychological and behavioural outcomes. Waiting for surveillance could introduce uncertainty, which was difficult to tolerate if patients were worried about having LC. When there was trust and confidence in HCPs, which were also regarded as reasons that influenced patients’ reactions, participants felt relief and reassurance and were motivated to attend surveillance scans. However, consistent with previous findings, some described significant anxiety and distress when they lacked understanding of what a nodule is, or lacked clarity about the rationale for monitoring over time rather than acting immediately.^26 27 34 35^ Lack of understanding seemed to be an outcome of patients’ experiences and to act as a factor that influenced their responses. Differences in how participants reacted to the indeterminate nodule and ensuing surveillance were influenced by the presence or absence of symptoms and comorbidities, how the result had been communicated and the terminology used and perceived support. Prior expectations for their lung health and whether their preferences for communication with HCPs (including primary care) were aligned with the communication they received also explained their different reactions.

While clinically significant adverse psychological responses have been previously reported to be relatively short-lived in lung and other cancer screening studies of indeterminate results,^13 36 37^ our study suggests the duration of worry and subclinical distress may be longer for some, particularly those whose first surveillance scan is indeterminate and therefore wait longer for repeat scans. Indeed, increased waiting times to receive results or to attend repeat scans, coupled with ineffective lay descriptions or nodule terminology that is interpreted to be concerning (eg, ‘abnormal’), led participants to feel concern and fear about their lung health.^27 38^ Research should seek to examine how adverse emotional reactions can be prevented through patient-centred approaches to communication. More specifically, participatory approaches with under-represented groups could be used to co-design and test lay explanations of nodules and the rationale for surveillance and methods to communicate these verbally for those identified as being more likely to experience distress.^27^

The present findings suggested that those who currently smoke or have a family history of cancer can have a greater propensity to experience distress and worry in response to an indeterminate finding.^36^ However, this relationship appears complex, as some participants who expected an abnormal finding due to their smoking history or comorbidities appeared to be more psychologically prepared as a result and felt relieved that their finding was less serious than LC and reassured that they were being closely monitored by HCPs.^39^,^41^ The psychological benefit of preparing individuals for the possibility of indeterminate nodules being detected before they participate in LCS has been suggested previously,^14^ in helping reduce concern for those receiving this result. Similarly, previous research in the incidental pulmonary nodule context suggests that well-intended anxiety-management strategies which avoid mention of cancer are ineffective and counterproductive.^23^ Instead, open communication about the low possibility of LC, emphasising early detection and building patients’ confidence in the effectiveness of surveillance, could appropriately manage anxiety, improve knowledge and adherence to repeat scans.

Increasing involvement of primary care as an adjunct tier of support is necessary, and a clear understanding of why HCPs’ level of concern was low appeared to have a positive impact on patient outcomes. Misalignment between patients’ preferences on means of disclosing and communicating nodule results, and actual nodule communication they received from HCPs, seemed to affect their cognitive and emotional reactions. Recent UK findings highlighted high satisfaction and patient preference for receiving nodule results via a letter. In that study, the majority stated their preference for a letter over verbal or in-person communication, which also influenced their experiences and responses. However, qualitative data from US LCS emphasised participants’ dissatisfaction with receiving results letters.^24 42^ In the current study, letter-based approaches appeared to be acceptable, but some highlighted the need to improve the way nodules were explained in the letter. Brief, concise information seemed to reassure some participants, yet too little information or a lack of clarity about what a nodule is and its severity appeared to limit their understanding about the surveillance process and increase uncertainty and distress over what followed, particularly when they were also unable to ask HCPs further questions. This reflects findings from cervical and breast cancer screening research.^24 26 43^ As described in prostate cancer screening research, patients with indeterminate pulmonary nodules may need active surveillance instead of ‘watchful waiting’,^44^ and they should be able to know and understand what the nodule and surveillance process are.^45^

Interestingly, the differences in nodule communication in the context of targeted lung health checks and how this might affect patients’ experiences and reactions could be explained by the way the letters are phrased and circulated by the different sites. Although there is a standardised letter template, this can be adapted by each site. Signposting reliable quality-assured information and support also appeared to be important for patients.^45^,^47^ Communication strategies ought to be targeted and tailored to those populations that are seldom engaged in cancer screening while considering cultural beliefs and racial/ethnic differences,^48 49^ as well as to those with comorbidities. Improving the phrasing of disclosing nodule findings and explanation of waiting times could reduce patients’ ambiguity and emotional distress.

The present findings suggest that effective information exchange between the patients and HCPs, as described by Slatore and Wiener,^23^ and additional information in lay language appeared to be important in explaining the results and outlining what the surveillance process involves. Brief consultations, in person or over the phone, with HCPs were crucial to decrease LC-related distress and worry and increase knowledge and understanding of screening risks and benefits.

### Limitations

Study limitations include the lack of participants from an ethnic background other than White, which prevents any insight into differences in experience in those from different ethnicities. Furthermore, despite efforts to identify potential participants through the sites and with incentives to participate, the study was unable to recruit participants with an unfavourable outcome following the initial surveillance LDCT. More women rather than men were recruited, which perhaps should be considered when interpreting the findings at a population level. Some participants, particularly men, may have been less willing to openly share their thoughts and feelings about undergoing surveillance. This could be interpreted in line with gender stereotypes that men implicitly hold beliefs that they need to look strong,^50^ and they have not been negatively impacted psychologically and emotionally by unpleasant experiences.

Data collection was carried out remotely, which may have negatively impacted what participants were willing to share with the researcher, and the researcher could not observe any body language. Although efforts were made to build rapport with participants, interviews are not natural conversations, but instead were being audio-recorded for research purposes, which may also have adversely affected participants’ openness. Although efforts were made to invite eligible individuals within 4 weeks of receiving their surveillance scan results, some participants were not interviewed until approximately a further 4 weeks after receiving the invitation because of unforeseen circumstances (eg, industrial action). Due to this delay, participants may have had difficulty recalling details, experiences or responses.

Lastly, we acknowledge that although an inductive analytical approach was taken, the interpretation of themes is likely to have been influenced to some extent by the use of the communication framework. It is also important to reflect on the characteristics of the researchers and the research context. The researcher who conducted the interviews was younger than the participants, had no experience of any serious respiratory illness (although this was not disclosed to participants) and approached participants as a university-based researcher. It is also possible that participants may have been less keen to honestly discuss any challenges they faced in clinical settings due to knowing the study-related collaboration between the researcher and the NHS Trusts. Nonetheless, this is the first study of its kind to explore experiences and psychological responses of patients undergoing LDCT-detected nodule surveillance.

## Conclusions

This multicentre qualitative study provided evidence that perceived support and effective communication with HCPs were instrumental to patients’ experiences and psychological responses to undergoing surveillance for a screen-detected nodule. Although trust in HCPs and current communication practices appeared to be acceptable to some participants for disclosing and communicating nodule results, there is a need to improve the way nodules and the surveillance process are explained to patients using clear, lay language. Refinement in the means of nodule communication could decrease LC-related distress, concerns and uncertainty, and also increase perceived reassurance, improve patients’ knowledge and understanding of nodules and psychological preparation for the possibility of surveillance.

## Supplementary material

10.1136/bmjresp-2024-002498 online supplemental file 1

10.1136/bmjresp-2024-002498 online supplemental file 2

## Data Availability

Data are available on reasonable request.
