# Supplementary material for: Patients’ experiences of, and psychological responses to, surveillance for pulmonary nodules detected through lung cancer screening
Source: BMJ Open Respir Res. 2025 Jun 12;12(1):e002498. doi: 10.1136/bmjresp-2024-002498 (PMC12164620; doi:10.1136/bmjresp-2024-002498)
Supplement: online supplemental file 1 [file bmjresp-12-1-s001.pdf]

Queen Mary
University of London

Interview Schedule

Understanding patient experiences of having further scans after a Lung Health Check

1. Warm-up Questions

Q1a: Have you ever taken part in an interview as part of a research study?

Q1b: Before having been invited for a Lung Health Check appointment, how would you describe your understanding of this Lung Health Check?

2. Pre-Lung Health Check Appointment

Q2a: Can you remember receiving your invitation to the lung health check appointment?

3. Time Between LHC Appointment and Disclosure of the Result

Q3a: Can you describe your experience of your first lung health check (LHC) appointment?

Q3b: Can you talk me through your experience while waiting to receive the result?

Q3c: Can you share your thoughts and feelings about when you found out that you had a small area of abnormality (lung nodule) and needed another scan?

Q3d: How much did you understand about the result?

Q3e: What kind of information did you receive from the healthcare professionals? (feelings and reactions)

Q3f: What kind of support did you receive from the healthcare professionals? (feelings and reactions)

Q3g: What do you think about the way your result was communicated to you by healthcare professionals and other staff you may have spoken to?

Q3h: What do you think they may need to do differently for the benefit of future patients?

4. Time Between Having Obtained the Result and Being Invited to First Pulmonary Nodule Surveillance Scan (Pre-Surveillance)

Q4a: Can you talk me through your thoughts and feelings of waiting for the first lung (nodule) scan of the small abnormal area that was found?

Q4b: How did you find that period of waiting?

Q4c: Can you remember receiving your appointment invitation to your first lung (nodule) scan for the small abnormal area?

Q4d: Can you describe your experience of having your first lung (nodule) scan for the small abnormal area?

Q4e: Can you describe your experience of finding out the results of your first lung (nodule) scan for the small abnormal area?

5. Post-/Under Surveillance

Q5a: Can you share your thoughts on the way the lung (nodule) result of a small abnormal area was communicated to you?

Q5b: What do you think about the ways that healthcare professionals and other staff involved in the Lung Health Check service communicated the result to you?

Q5c: What do you think they may need to do differently (or keep doing) for the benefit of future patients? How do you believe future patients should be treated?

Q5d: Has there been any specific moment on this journey (from when you first received your invitation to the lung health check appointment up until now) when you felt like you were under pressure? Why?

6. Closing Questions

Q6a: Would you mind me asking if anyone brought up smoking with you at all?

Q6b: Can you share your thoughts about being under surveillance during the COVID-19 pandemic?

Q6c: Finally, I would like you to talk about how you feel about your lung health now?

Q6d: Thinking ahead, is there anything you would like to change about your lifestyle or health?

7. Close and Debrief

Q7a: Is there anything else you would like to mention that you feel we have not covered?
