# Supplementary material for: Patients’ experiences of, and psychological responses to, surveillance for pulmonary nodules detected through lung cancer screening
Source: BMJ Open Respir Res. 2025 Jun 12;12(1):e002498. doi: 10.1136/bmjresp-2024-002498 (PMC12164620; doi:10.1136/bmjresp-2024-002498)
Supplement: online supplemental file 2 [file bmjresp-12-1-s002.pdf]

Table 2 (cont.). Selective illustrative quotes for theme descriptions

Quote no	**Reactions and outcomes**
	Psychological harms and benefits
Q1	*"Frightened, I suppose, because I don't know, it's a stupid thing to say, but once I'd got the results of the second scan and they'd told me what the plan of action was right now, you're going, this is what's going to happen, and it did, and I was scared and still in disbelief because I kept thinking no, no, no, no, no, they've got me mixed up, are you sure you haven't got me mixed up with somebody else?" (F/FS)*
Q2	*"... I did worry after the third scan, there was an abnormal area, then I was worried for three months. ... I got very stressed. I got very stressed, very worried, I start researching about it. I got very, very worried about it. ... Yeah, cancer, I'm dying. ... Yeah, absolutely. I spent three months worried. I'm saying three months because that would be the next time I was going to have another scan. ... Yeah, because this is a big blow. It's a big worry. Although it was myself, I decided to go for the test as a volunteer just out of, just to get it checked, but it did affect me, big time. It's hard, really, and there is an uncertainty that I have lived for, since the third scan, no, the second scan, right? I had three scans altogether, so the second scan, so the third one was very uncertainty and now more uncertainty for the next year until I have the other one. It's like you're living in a very big uncertainty, you don't know what is going to happen. I don't know, it's horrible, really." (F/CS)*
Q3	*"and I used to go every couple, I think every couple of months, I'm not sure, and I thought, how nice that they are keeping an eye on me, and as I said, I did enjoy going, and that's the reason, but as I said, I've never, never thought any more of it because as I said, they were keeping a check on me and going every couple of months to have these checks done and I did look forward to going." (F/CS)*
	Change in cancer risk behaviours
Q4	*"Not really. I have a pair of lungs. They workish. I don't cough. I now find it quite difficult to walk up the escalator at a tube station. I get out of breath more easily than I used to before. I am aware of that. But I can still swim, I still dance*

	without any problem, without getting breathless. I'm fine, I think, for my age" (F/CS)
Q5	*"By looking after myself, I stopped smoking when the first one, when I got the phone call, so I changed my diet and started looking after myself a bit more, bit more health wise." (F/FS)*
Q6	*"Well, I feel relieved that I'm going to have another appointment next year. And I do worry because I can't give up smoking especially now knowing there's a nodule, I should be trying even harder but I just can't seem to do it. But the way I look at it is at least every year I'm having this test." (F/CS)*
Lack of knowledge and understanding about the surveillance process	
Q7	*"Yeah, I, I'm concerned about this delay, that it might not be good news. I'm, I find the longer I'm waiting for the results, the more negative I feel about it. Not the whole experience of taking part in the lung health check, but of the second scan and what they may have found. I've found that negative. If I'd have had the result by now then I've had, obviously it was, you'd know what you're dealing with but I don't know what I'm dealing with at the moment because I haven't heard from them." (F/FS)*
Q8	*"Oh, I didn't really understand anything at all. I don't, it's not my expertise. I don't know. All I knew was that there was something there. I wasn't even sure, because I'm old school. I don't understand millimetres and things like that. To me, you might just as well be talking, I don't know, carrots or something. It doesn't mean anything to me. So I didn't, I wasn't even aware of how big it was, where it was, or, although it's written in there how big it is and where it is, I didn't understand it. ... I wouldn't have understood very much of it at all. So I probably would have phoned up who I got the letter from. I probably would have phoned them up and said, well, do I need to worry about this? Is it anything to worry about?" (F/FS)*
Q9	*"Yeah, and then the other letter then with the results was just to say the CT scan of your lungs has shown a small spot called a nodule. So of course the minute you read that there is something on your lung then you do obviously start to think, oh my goodness, and then, as I say, in the letter it says, this is a common finding on scans and often nothing to worry about etc. Please be assured that this does not require immediate follow up, further investigation but we'd like to*

	see you in 12 months. So there was no real explanation as to what this nodule is. There was a booklet I believe with it, but that doesn't give you particularly a chance to ask, you can't ask a booklet a question. So the, not a great deal of explanation as to what causes it and you are left a little bit up in the air, which is why, as I say, then you start Googling things." (F/FS)
Q10	*"I did ask my GP. You know, have you had a letter back from [service name] and they said, yes, nothing to worry about. I saw it on the computer. No cancer. And I suppose that word you think, well that's the worst word you can hear. I didn't really think much about anything else."* (F/FS)
Q11	*"But when I sat and read it, the first thing I did was I texted my [family member]… She got straight back to me and she got me to read it all out to her, and she told me exactly what it all meant. Therefore, I wasn't too worried because she said to me, that could have been there, she said, for a long time. She said, they want you to have another one, she said, to follow it up. She said, so let's just wait and see what happens when they do the second one and see what the results are. So you see, although I got a little bit anxious about that, I got in contact with her straight away, and she reassured me … And that was my reason for phoning her, because she is very good at explaining things in my language. She knows me very well, and she can explain things to me so that I understand. So I'm very lucky in as much as that I've got her."* (F/FS)
Q12	*"Well, it was a bit scary because my [significant other] had been diagnosed with lung cancer and then to be told that I've got a nodule as well. It was a bit frightening to think that we might both have cancer. It was a bit scary. It was a bit scary."* (F/FS)
Q13	*"I did discuss that with, as I say, my [significant other] and my [significant other] and it's a usual thing when you're talking, you're going for the scan and hopefully everything was going to be OK. … we spoke in depth about the cancer that she had, and obviously hers was far more serious than what's, the news that I had in a sense, because I, as I say, we talked about it and you get a little bit of confidence, because you've seen somebody that has already had the issue with cancer and had an operation and sh' was, she is fine."* (M/FS)
**Modifying influences on psychological and behavioural responses**	

Trust, communication and terminology affected reassurance and understanding	
Q14	*"At that time. I can, if I've got any other questions, the consultant, when I got called into the hospital to see him, he asked me a few times if I've got any questions, and I just kept saying no, because nothing come to mind. But he said, if you do think of something, don't hesitate to phone my secretary and I'll phone you back, or come and see me. But, no, I haven't worried about it. I just leave it. He didn't seem that concerned, so I thought, well, if he doesn't seem that concerned, and I don't have to go back until another 12 months." (F/CS)*
Q15	*"Well I don't see that there's a problem with having the scans done, but as I say I'm all in favour of these sorts of things. So it's flagged up that I've got a problem, a slight problem with my lungs and I'm aware of it so that I know what to do, if I get a sudden chest infection I know exactly what I've got to do and what, and how to, so does my GP which is a good thing, so I'm quite happy with that." (F/FS)*
Q16	*"I've had two letters where I've got nodules. So the one before, the first one I got I nearly passed out. I thought, what the hell? I quickly rung them because there was nothing to say, we've spotted it, it's this big or whatever, nothing. Just a nodule or an abnormality on your lung." (F/FS)*
Q17	*"Yeah, yeah. I still don't understand what it actually means, you see, so I don't know whether I'm going to have another scan or, I just don't know what to do. ... Yeah, if somebody yeah, could, that would be the question I'd ask if they could tell me exactly what it means or how bad it is." (F/FS)*
Q18	*"When they said small anomaly, it's a bit ambiguous. I suppose they're trying not to scare people by saying, we found something that needs further investigation. If I didn't know that I always had PED, PF, I would have been very scared, but because I knew I had PF, so it didn't scare me so much. As long as it, that letter didn't say oh, a cancer thing has shown up, that, I was fine." (F/FS)*
Absence of symptoms and awareness of early detection affected nodule perceptions	

Q19	*"It was just that something had been found and actually I was quite relieved because I thought well, if there is anything there it's because I've got no, I'm not wheezy, I don't cough. I don't bring anything up. Apart from the fact that now I do get very, very breathless but at the time I had no symptoms at all. So I actually found it, that if there was anything untoward someone was aware of it and was keeping an eye on it. So yeah, so no it was fine."* (F/FS)
Q20	*"But given they didn't find anything the first, no I, as soon as they didn't find anything the first time, I was kind of well anything they find from now on is going to be really early spotting of any problem and therefore obviously with any tumour or cancer or anything like that, then early detection is absolutely key. So I felt in good hands. I didn't really worry about it to be honest" (F/FS)*
Preferences for communication and support from trusted others and HCPs within and outside of the screening team	
Q21	*"No, I, actually I thought it was very, very clear and it, I knew exactly what they were saying in the letter. There was no misunderstanding, I knew exactly. Actually, it was quite a very good letter explaining what was wrong, what they found and then the follow up scan. So, yeah, I was quite impressed with the letter. ... No, because obviously receiving the letter, I was relieved, so I thought there was no reason to phone them up again."* (M/CS)
Q22	*"The letter was quite explanatory. ... I quite understood what they were saying, yeah. ... Very reassuring and yeah. Peace of mind"* (M/CS)
Q23	*"I think it seems to work well. The letters are short and when you have the appointment you don't feel rushed, you feel people are there to answer your questions if you've got them. I don't really see what more you can do." (F/FS)*
Q24	*"Not much. It wasn't very clear really. The letter wasn't really very clear. I mean it said a small nodule, I wasn't sure what that meant in the, I can't remember if it was in the left or the right lung at the, near the base. I, I'm not sure, it wasn't very clear really. Well I didn't interpret it as anything in particular, I wasn't sure what it could be."* (F/FS)

Q25	*"I didn't find it was explained properly. Because first when I heard it was a nodule or whatever, and then it was abnormality, I thought, oh, what's that? So yeah, it wasn't explained properly." (F/FS)*
Q26	*"I've been waiting quite a while now for my results of my last, of my second scan. I still haven't heard anything, I'm getting a bit, I need to know now. I need to be told if there was anything, if that nodule has disappeared or if there's been any chances, I need to know. I need to know and I haven't been contacted. I think this is a long delay and it's not very good. It's not good at all, this long delay. ... But this, then I had the second one because they found something, and it's the second, it's the results of the second scan I, I'm not happy with because I still haven't heard, I still haven't had a letter or had a phone call. So I'm left in limbo not, worrying now if they've found anything again on the scan. Because it's got me worrying about things now. Could it be a small cancer tumour? Could it be, you know, I'm just worried now, I'm getting worried." (F/FS)*
Q27	*"I haven't done to be fair because that was, that letter was ... when they said that they would see me in 12 months. So I haven't done anything about that yet, but is on my mind as to why I've got to wait 12 months. So I could make a phone call, I could ring them and say that I'm not happy about a 12 month wait, because, as I say, it is something that does play on my mind a bit." (F/FS)*
Q28	*"OK. I mean who can I contact? The GP? If they had the result they would just tell me what was in the letter and there was no point in contacting the [service] because, again, they can tell me nothing but what was in the letter. So I was just fingers crossed and waiting for the second appointment, or results to come. Because the next, just, x-ray was going to be of MRI was going to, not MRI it's the other one isn't it? CAT scan. It's going to be in a few months I just thought I had to get it out of my head and just wait for the next X ray." (F/CS)*
Q29	*"They were concerned, but they were supportive, and I think I spent most of my time reassuring them, as opposed to the other way." (F/FS)*
Q30	*"Yeah. I would much rather have had that phone call than received a letter because the phone call was someone there who was calm, explaining everything to me, put me at ease rather a letter, I would've been on the phone to all kinds,*

	trying to find out what's going on and stuff like that. So the actual phone call was far better. ... All right. Just get the letter saying we found something and come back in two weeks' time, that would, I think that would have been a bit cruel. That's why I'm saying the phone call was much, much better. Although it made me worry, but it was much, much better" (M/CS)
Q31	*"I mean it would be nicer to get the result over the phone by somebody because then, if there is a problem, they can explain it and maybe relieve you a little if it's not too serious. And then whilst you're on the phone you might, while still on the phone you might remember, like think of questions to ask."* (F/CS)
Q32	*"So I think from the second scan just to receive a letter basically to say what the result is and don't, more or less saying, oh you don't need to worry, I think there should really be more support and a bit more explanation. You get a booklet but I think sometimes you need perhaps a telephone call or chat with somebody, so it's more personal, not just you're just another number. This is, oh this is a bit, it's sort of normal, don't worry about it type of thing, we'll see you in 12 months. That isn't their attitude, don't get me wrong because NHS staff aren't like that, but it's not personal. So I think that perhaps would have been nicer to have had a chat about it, which is obviously what your research is doing, it is a personal conversation that we are having, you are asking me how do I feel, what are my thoughts? So in a way without your caring it just makes you feel, well this is it, we found that, don't worry about it, we'll see you later."* (F/FS)
Q33	*"I think what would be preferential to me would've been, yes the letter of their findings and an invitation if I would like an appointment to see the specialist, but you don't get that ... I would say, when I was there, obviously there was couples there. There was disabled people with a carer there. I believe some form of information or a leaflet regarding whatever symptom you've got regarding your lung can be passed over to you and say look this is what it's all about, this is what we can do for you and please read it and any concerns as to go to your GP or make an appointment with the specialist. That's how I would see it, but I didn't get anything."* (M/CS)

Q34	*"But because to me then it shows that the department that's actually done it has done what they've got to do and just say shoved it back to the management of the hospital and said, here you are, send him a letter, and then that normal number comes up and you ring it and for the department you want, their stupid, that phone, God no. So I wouldn't bother. ... I think really the main event is from talking to you about all this is how the GP, all GPs, should show more concern when they receive notification from hospitals that your patient has had this done and what's come back is this. Even if it's from his receptionist, just a reassurance to let you know that things have come back from the hospital and everything's OK, you've got nothing to worry about or we've had things back from the hospital from your, the doctor would like to see you, can we make an appointment? Fair enough. They could play a far bigger role helping the NHS in all departments do what they're doing." (M/CS)*
Q35	*"If they sent the results to the patient's GP I think the onus should be on the GP to call his patient in or the GP can ring the patient up and say, I see you've had a scan done. I've been informed by the results, I've seen it myself and everything's fine. Or I'm afraid we're going to have to make you another appointment due to, you can get that reassurance from your own GP but you, the GPs today are, just aren't interested ... The GP wants to know, but when he gets it he just files it away. And I think that that's wrong, your GP should contact his registered patient and I think that's where the stop gap is. Everybody knows their own doctor. So, therefore you've got that trust, that confidence between the two of you, rather than somebody from the hospital who you've never met before, she doesn't know you, doesn't know that, giving you a leaflet, and it's blah, blah, blah" (M/CS)*
Existing physical comorbidities and self-reported mental health conditions	
Q36	*"... because I was already in the stage of, I've got COPD, so I knew there was a problem with my lung ... Because I do suffer, I suffer with this breathlessness and sometimes it's as if I've got no oxygen in my body, that type of a thing." (M/FS)*
Q37	*"No, because my chest, although I've been labelled as mild COPD for a number of years and I think that's just going on smoking history, because the lung*

	volume was at, I know, I have a diploma in asthma and COPD and God knows what else, but in the UK, 70% lung volume is perfectly normal.” (F/FS)
Q38	*“I have only fairly recently given up smoking after a very long time, and the fact that I've also got certainly a mild form of COPD anyway diagnosis, those three things combined make me much more aware of what's going on with my chest ... I had Covid last October and was quite poorly for two weeks but wasn't hospitalised. And since then, my breathing has been a little bit laboured, but it is getting better.” (F/FS)*
Q39	*“I am worried but there's nothing I can do about it. They say it's a common thing, that the risk is very low, but there's something there. They say that the risk is, it is up to 5%, that's a very common thing to have, blah, blah, blah, but yeah, I'm worried, because I did have breast cancer.” (F/CS)*
Q40	*“unfortunately for me I've been suffering from depression and anxiety. So that didn't help one bit, you know what I mean? So what, it made it a lot worse, a lot more difficult for me to take in, because that just added to my depression and anxiety, if you're with me? ... No. I never called the GP or anything like that. After discussing it with my wife, I felt a bit better but I was still very apprehensive of what they might find.” (M/CS)*
Q41	*“I think I did express that I'm a very nervous person. I've got, I'm on antidepressants, I've got PTSD. So, yeah, I think they recognised in me that I was nervous, and they didn't say anything really bad. ... Obviously I smoked for 40 years. I said, I assumed something was going to be on there. I think I was so happy it wasn't cancer that I didn't care.” (F/FS)*
	Pre-existing expectations for nodule results
Q42	*“Well, when I look at my previous lifestyle, like I said, when I was younger, I was a really heavy smoker, so I anticipated that had caused some damage to my lungs because of that activity. So, I was, I didn't need anything explaining. I knew what was going on ... I was a little, like I say, I've been a heavy smoker and heavy drinker in the past, so I'm expecting there to be some damage as a result of that. So, I wasn't unduly nervous or anything. I was just curious as to why I needed to go back and later on after, they finally told me why, which was that they'd seen a third nodule. But I wasn't upset or anything, but I was, knew what was going on.” (M/FS)*

Q43	*"Obviously I smoked for 40 years. I said, I assumed something was going to be on there. I think I was so happy it wasn't cancer that I didn't care." (F/FS)*
Q44	*"I used to work in the coal mining industry for most of my working life, and we used to have our chest x rayed every two or three years anyway. So, I was pretty sure what was going on." (M/FS)*

Note. Participant codes (e.g., M/CS) represent gender (i.e., M = Male; F = Female), and smoking status (i.e., CS = Currently smoking; FS = Formerly smoked; NS = No smoking).
